# GABA ameliorates diabetic kidney disease-induced podocyte injury via JAML-FPR2 axis-mediated suppression of macrophage infiltration

**DOI:** 10.1080/0886022X.2026.2736906

**Published:** 2026-10-01

**Authors:** Yibo Zhuang, Chenlu Fu, Zheng Guo, Yan Xu, Jun Zhou, Huiping Ni, Aihua Zhang

**Affiliations:** ^a^ Jiangsu Key Laboratory of Early Development and Chronic Diseases Prevention in Children, Nanjing Medical University, Nanjing, China; ^b^ Department of Pediatrics, The First People’s Hospital of Changzhou, The Third Affiliated Hospital of Soochow University, Changzhou, Jiangsu Province, China; ^c^ Respiratory Medicine, The First People’s Hospital of Changzhou, The Third Affiliated Hospital of Soochow University, Changzhou, Jiangsu Province, People’s Republic of China; ^d^ Changzhou medical Center, Nanjing Medical University; ^e^ Department of Nephrology, Children’s Hospital of Nanjing Medical University, Nanjing, China; ^f^ Nanjing Key Laboratory of Pediatrics, Children’s Hospital of Nanjing Medical University, Nanjing, China

**Keywords:** GABA, DKD, JAML, FPR2, M1 polarization, apoptosis

## Abstract

This study demonstrates that gamma-aminobutyric acid (GABA) ameliorates diabetic kidney disease (DKD) by modulating macrophage-driven inflammation and podocyte injury through the JAML/FPR2 signaling axis. In streptozotocin (STZ)-induced DKD mice, GABA administration significantly improved renal function by reducing serum creatinine, urea nitrogen, 24-hour urine protein, attenuated glomerular hypertrophy/mesangial expansion, and suppressed pro-inflammatory cytokine production (TNF-α, IL-1β, iNOS) in renal tissue and serum. GABA inhibited glomerular macrophage infiltration (CD68^+^ cells) and M1 polarization while mitigating renal apoptosis and podocyte injury by restoring nephrin and podocin. In a high glucose (HG)-stimulated macrophage-podocyte co-culture model, GABA reduced HG-induced podocyte apoptosis in a macrophage ratio-dependent manner by reversing M1 polarization and inflammatory cytokine overproduction. Mechanistically, GABA normalized DKD-elevated JAML expression in renal tissues and podocytes, while JAML overexpression abolished GABA’s renoprotective effects by reactivating inflammation, macrophage recruitment, and podocyte apoptosis. Co-IP confirmed JAML interacted with receptor formyl peptide receptor 2 (FPR2), which mediated DKD-driven macrophage infiltration, as FPR2 knockdown abrogated JAML-induced CD68^+^ cell accumulation. Collectively, GABA alleviated DKD progression by disrupting the JAML/FPR2 axis to suppress macrophage-mediated inflammation and podocyte injury, highlighting its therapeutic potential for diabetic kidney disease.

## Introduction

Diabetic kidney disease (DKD), a prevalent complication of diabetes mellitus, affected approximately 30% of individuals with type 1 diabetes (T1DM) and 40% of those with type 2 diabetes (T2DM) globally [1]. Recognized as a leading contributor to end-stage renal disease worldwide, DKD presents significant clinical challenges compounded by the substantial financial burden associated with renal replacement therapies such as dialysis and kidney transplantation [2]. This pressing reality underscored the critical need for innovative therapeutic strategies to effectively halt disease progression and mitigate its socioeconomic impact.

Previous studies have demonstrated that inflammatory responses mediated by cytokines—including TNF-α, IL-1β, IL-6, and iNOS, which are predominantly secreted by activated M1-type macrophages—along with fibrosis and apoptosis, collectively drive the progressive decline of renal function in DKD through mechanisms involving inflammatory cell infiltration and interstitial fibrogenesis [3–6]. Specifically, upon activation, M1 macrophages release substantial amounts of early core inflammatory factors such as TNF-α and IL-1β. These cytokines not only directly injure podocytes and disrupt the filtration barrier but also further activate downstream signaling pathways such as NF-κB, establishing a self-amplifying inflammatory loop [7]. Concurrently, iNOS, a key marker of M1 polarization, is upregulated and leads to excessive production of nitric oxide (NO), exacerbating oxidative and nitrosative stress in the kidney [8]. Moreover, studies have confirmed that hyperglycemia and angiotensin II synergistically upregulate renal TLR4 expression, thereby enhancing the activation of the NF-κB pathway and creating a positive feedback loop that promotes the release of critical inflammatory mediators such as TNF-α, IL-6, and IL-1β [9,10]. Renal macrophage infiltration, through tubular‑macrophage crosstalk, not only perpetuates macrophage activation but also directly induces tubular epithelial injury, thereby aggravating the pathological progression of DKD [4]. In this process, the imbalance between M1 and M2 macrophage phenotypes plays a pivotal role: the M1‑dominant pro‑inflammatory state secretes IL‑12, TNF‑α, IL‑1β, and iNOS, whereas anti‑inflammatory factors associated with the M2 phenotype, such as IL‑10, are suppressed. This imbalance further deteriorates the inflammatory microenvironment in the kidney [11,12]. The importance of this mechanism is supported by multiple interventional studies. For example, in models of diabetic nephropathy, inhibition of iNOS or downregulation of TNF‑α and IL‑1β expression significantly alleviates renal inflammation, oxidative stress, and fibrosis, while improving podocyte structure and function [7,13,14]. Together, these findings highlight the central role of macrophage polarization and the key inflammatory factors they secrete in the progression of DKD and underscore the therapeutic potential of targeting this inflammatory network. Therefore, monitoring the expression levels of TNF‑α, IL‑1β, and iNOS not only provides an accurate assessment of the extent of macrophage‑driven inflammatory injury in DKD but also offers a direct and reliable molecular basis for investigating whether GABA exerts renoprotective effects by modulating this critical pathway.

Emerging evidence elucidated γ-aminobutyric acid (GABA) had anti-inflammatory properties as a modulator of immune cell-driven inflammatory pathways. Notably, GABA administration significantly attenuated pathological inflammation in murine models of rheumatoid arthritis and non-obese diabetic syndromes [15,16]. Mechanistically, GABA-salt suppressed macrophage M1 polarization and ameliorated diet-induced hypercholesterolemic/hypertensive conditions [17]. Empagliflozin-GABA combinatorial therapy preserved β-cell mass and ameliorated glucose intolerance in STZ-induced diabetic mice [18]. In Mongolian sheep-derived renal cortical cells, GABA counteracted hyperglycemia-induced oxidative injury through transcriptional upregulation of antioxidant defenses and metabolic homeostasis modulation [19]. Building upon these mechanistic insights, we systematically investigated GABA’s nephroprotective potential in streptozotocin-induced diabetic kidney disease murine models and macrophage-podocyte communication in HG environments.

Emerging research had highlighted the pivotal regulatory functions of junctional adhesion molecules (JAMs), immunoglobulin superfamily members expressed across leukocytes, platelets, and epithelial/endothelial cells, in orchestrating immune activation dynamics, inflammatory cascades, and cellular architectural processes, including polarity establishment, epithelial barrier maintenance, and leukocyte transendothelial migration [20,21]. Junctional adhesion molecule-like protein (JAML), a recently characterized component of the JAM family, exhibited broad cellular distribution spanning both innate and adaptive immune effector populations such as monocytes, neutrophils, and memory T lymphocytes, as well as select parenchymal cell lineages, positioning it as a critical molecular interface in immunoregulatory processes [22]. Emerging evidence from macrophage-focused investigations had elucidated novel immunoregulatory mechanisms of JAML, establishing its dual role as both a pathogenic mediator facilitating acute kidney injury progression and a clinically relevant biomarker for renal damage severity [23].

In this article, we aimed to elucidate whether GABA was involved in regulating STZ/HG-induced podocyte injury, directing the macrophage polarization, altering the inflammatory process (pro-inflammatory reversal to anti-inflammatory), proving the effectiveness of GABA in DKD, and further exploring its potential mechanisms.

## Materials and methods

### STZ-induced diabetic kidney disease (DKD) model

Eight-week-old male C57BL/6 mice, weighing between 20 and 25 g, were purchased, reared under specific pathogen-free conditions, and randomly divided into four groups (*n* = 5/group): the control, control + GABA, DKD, and DKD+GABA groups. DKD mice were established *via* five-day intraperitoneal injections of 50 mg/kg streptozotocin (HY-13753; MedChemExpress), while controls received equivalent volumes of citric acid buffer. Following a 6h fasting period, tail vein blood glucose levels were measured one week post-intervention using a glucometer, with mice exhibiting fasting glucose ≥16.7 mM classified as diabetic. GABA (A5835; Sigma-Aldrich) was administered orally to control/DKD mice at a dose of 10 mg/kg daily for 8 weeks. These DKD mice were divided into DKD+OE-JAML, DKD+KD-FPR2, DKD+OE-JAML+NC, and DKD+OE-JAML+KD-FPR2 groups. NC, OE-JAML, and KD-FPR2 (1x10^7^ TU/mL, 100 µL) were injected into DKD mice *via* the tail vein. All experiments involving animals were conducted in accordance with the ARRIVE Guidelines 2.0 and were approved by the First People’s Hospital of Changzhou (Ethical Number: 2024-KD-205). Mice were euthanized *via* intraperitoneal injection of sodium pentobarbital (150 mg/kg). Death was verified by cessation of vital signs, consistent with AVMA guidelines.

### Cell culture

All *in vitro* experiments were performed with three independent biological replicates. Conditionally immortalized mouse podocytes (MPC-5) were cultured in low glucose (1 g/L) DMEM (10567022, Gibco) supplemented with 10% FBS (16140071, Gibco) at 37 °C with 5% CO_2_, which was defined as a normal glucose (NG) environment. A high-glucose (HG) environment was defined by the addition of 3 g/L glucose to NG medium [24]. The murine macrophage cell line RAW 264.7 (Procell, Wuhan, China, Cat. No. CL-0190) was routinely maintained in high-glucose (4.5 g/L) DMEM (10566016, Gibco) supplemented with 10% FBS (16140071, Gibco) at 37 °C with 5% CO_2_. For all experiments, RAW 264.7 cells were switched to and equilibrated in the same NG or HG DMEM (as described for MPC-5 culture) for 24 h prior to any treatment or co-culture to ensure synchronization of the glucose environment.

### Measurement of creatinine, urea nitrogen, 24-h urine protein, tumor necrosis factor-α (TNF-α), inducible nitric oxide synthase (iNOS) and interleukin-1β (IL-1β) in serum

Mouse blood samples were centrifuged at 3,000 × g for 10 min (4 °C) to isolate serum, followed by biochemical analysis of renal function parameters using commercial kits: creatinine (sarcosine oxidase method, C011-2-1), urea nitrogen (C013-2-1), and 24-h urinary protein (C035-2-1) from Nanjing Jiancheng Bioengineering Institute, and macrophage-derived inflammatory markers (TNF-α/IL-1β, PT512/PI301, Beyotime; iNOS, SP14401, Wuhan Saipei Biotechnology Co., Ltd) quantified through enzyme-linked immunosorbent assay (ELISA).

### Hematoxylin and eosin (HE) and immunohistochemistry (IHC) assay

Renal tissue sections (5 μm) across experimental groups underwent HE staining through sequential immersion in Mayer’s hematoxylin (5 min), hydrochloric acid-ethanol differentiation (0.5% HCl, 30 s), and 1% eosin counterstaining (2 min), followed by ethanol dehydration, resin mounting, and histological documentation using Olympus BX53 optical microscopy (Tokyo, Japan). For glomerular area quantification [25], at least 10 intact, non-transected glomeruli were randomly selected from each HE-stained section using the same Olympus BX53 optical microscope. Glomerular boundaries were delineated based on morphological landmarks, including the distinct Bowman’s capsule contour and intraglomerular capillary loop distribution, by two independent researchers in a double-blinded manner (without access to sample grouping information). The glomerular area was measured using ImageJ software (version 1.8.0), and the average of the two researchers’ measurements was taken as the final glomerular area per sample to minimize inter-observer variability and ensure data objectivity.

Renal tissue sections underwent standard IHC processing, including thermal stabilization (65 °C/2 h), xylene dewaxing, gradient ethanol rehydration, and heat-mediated antigen retrieval in citrate buffer (pH 6.0), with subsequent 5% goat serum blocking (RT/1h) prior to overnight incubation (4 °C) with primary antibodies: CD68 (ab303565; 1:500; Abcam), Nephrin (ab216341; 1:500; Abcam), and Podocin (20384-1-AP; 1:400; Proteintech). Immunostaining was developed using HRP-conjugated secondary antibodies (RT/1h), YT8204 DAB substrate (Beijing Yita Biotechnology) with 5-min chromogenic development, hematoxylin counterstaining, and resin mounting, followed by CD68^+^ cells in glomerulus quantification in five random fields.

### Apoptosis measurement

In a macrophage/podocyte co-culture system using polycarbonate insert cell culture devices, we seeded 2 × 10^5^ podocytes in the lower chamber and 2 × 10^5^ macrophages (NG, Mannitol, HG, HG+GABA, HG+Mφ-1R (Mφ: macrophage, R: ratio; macrophage-to-podocyte ratio 1:1), HG+Mφ-2R (macrophage-to-podocyte ratio 2:1), HG+Mφ-4R (macrophage-to-podocyte ratio 4:1), or HG+Mφ-4R + GABA-treated) in the upper chamber, followed by 48-h incubation. Podocytes in each group were digested using trypsin without EDTA and then collected. Finally, in accordance with the instructions provided with the Apoptosis Detection Kit (Yeasen, 40302ES60), 1 × Binding Buffer was added to resuspend the cells, thus forming a cell suspension. Subsequently, 5 μL of Annexin V-FITC and 5 μL of propidium iodide (PI) were added for labeling. Finally, the apoptosis rate of the cells was analyzed by flow cytometry (Beckman Counter).

### Western blotting analysis

Proteins were extracted using RIPA Complete Lysis Buffer (P0039, Beyotime), quantified by BCA Protein Assay Kit (P0011, Beyotime), and then 20 μg protein solution was fractionated by 6% or 10% BeyoGel^™^ Plus Precast PAGE Gel for Tris-Gly System (P0449S or P0462S, Beyotime) before being transferred to PVDF. After blocking with 5% nonfat milk or BSA, the PVDFs were incubated with primary antibodies. The antibodies were listed as follows: anti-CD80 (66406-1-AP; 1:1000; Proteintech), anti-CD86 (13395-1-AP; 1:2000; Proteintech), anti-CD163 (ab182422; 1:1000; Abcam), anti-CD206 (ab300621; 1:1000; Abcam), anti-Nephrin (ab216341; 1:1000; Abcam), anti-Podocin (20384-1-AP; 1:2000; Proteintech), anti-iNOS (ab178945; 1:1000; Abcam), anti-JAML (ER61607; 1:1000; HUABIO), anti-FPR2 (30989-1-AP; 1:1000; Proteintech), and anti-β-ACTIN (66009-1-Ig; 1:3000; Proteintech). The membranes were washed and incubated with HRP-goat anti-rabbit secondary antibody (RGAR001; 1:5000; Proteintech) for 2 h at room temperature, then the protein bands were visualized using the SuperPico ECL Chemiluminescence Kit (E422-01, Vazyme, China) and subsequently analyzed with Image J software.

### Reverse transcription-quantitative polymerase chain reaction (RT-qPCR) analysis

Total RNA was extracted using TRIeasy^™^ RNA extraction reagent (TCM Free) (19202ES60, Yeasen), and reversed to cDNA using HiScript III RT SuperMix (R222-01, Vazyme), and qPCR was performed using *TransStart*^®^ Top Green qPCR SuperMix (AQ131-01, TransGen Biotech). Primers sequences used were as follows: iNOS (forward 5′-GAGCGAGTTGTGGATTGTC-3′; reverse 5′-CCAGGAAGTAGGTGAGGG-3′); JAML (forward 5′-CCATACTGAGGAGGAGACA-3′; reverse 5′-TTGAAGTTTGATTGAGCC-3′); TNF-α (forward: 5′‐GGCGGTGCCTATGTCTCA‐3′; reverse: 5′‐CCTCCACTTGGTGGTTTGT‐3′); IL-1β (forward: 5′‐GTTCCCATTAGACAACTGC‐3′; reverse: 5′‐GATTCTTTCCTTTGAGGC‐3′); and ACTIN (forward: 5′‐AATCGTGCGTGACATCAA‐3′; reverse: 5′‐AGAAGGAAGGCTGGAAAA‐3′).

### Co-immunoprecipitation (Co-IP) assay

Cellular lysates prepared in Cell Complete Lysis Buffer for Western and IP (P0037-100 mL, Beyotime) containing protease/phosphatase inhibitors underwent pre-clearing with Protein A/G agarose (P2197M, Beyotime), followed by sequential immunoprecipitation using 2.5 μg antibodies: JAML (ab183714; Abcam), IgG (6990; Cell Signaling Technology), and anti-FPR2 (30989-1-AP; 1:1000; Proteintech), ultimately yielding immunoprecipitation complexes for SDS-PAGE separation and immunoblotting with HRP-conjugated secondary antibodies.

### Immunofluorescence (IF) and terminal deoxynucleotidyl transferase dUTP nick end-labeling (TUNEL) staining

Following 24-h fixation in 4% paraformaldehyde at 4 °C, tissues were processed through paraffin embedding and transversely sectioned (4 μm) for immunofluorescence analysis, with sections sequentially incubated with anti-JAML primary antibody (21302-1-AP, 1:100; Proteintech), Multi-rAb^™^ CoraLite^®^ Plus 488-Goat Anti-Rabbit Recombinant Secondary Antibody (H + L) (RGAR002; 1:100; Proteintech), and DAPI nuclear counterstain (ID2250, Solarbio), followed by systematic image acquisition using an Olympus BX53 fluorescence microscope. SF488 TUNEL Cell Apoptosis Kit (T2196, Solarbio) was utilized to determine the apoptosis in kidney tissues.

### Statistical analysis

All results, obtained from three independent experiments, are expressed as means ± SD and analyzed using one-way ANOVA and *t*-tests with GraphPad Prism 7.0. *p* < 0.05 is considered statistically significant (*p < 0.05; **p < 0.01; ***p < 0.001).

## Result

### Effects of GABA treatment on renal function and structural damage in STZ-induced DKD mouse model

This study established a DKD mouse model through STZ administration and evaluated the therapeutic efficacy of oral GABA administration, with blood and 24-h urine samples collected following an 8-week treatment period prior to euthanasia. The DKD group exhibited significant elevations in serum creatinine, blood urea nitrogen, and 24-h urine protein levels compared to the control group, while GABA treatment (DKD+GABA) effectively attenuated these pathological markers compared to DKD group, and the control + GABA group showed no significant differences from the untreated control group (Figure 1A–C). The DKD group exhibited glomerular hypertrophy, mesangial thickening, and reduced renal capsule sac volume, all of which were significantly attenuated by GABA treatment (DKD+GABA group), with quantitative histopathology further confirming that GABA effectively reversed the DKD-induced glomerular enlargement (Figure 1D and E). In summary, these data confirmed GABA’s therapeutic efficacy in DKD and its good biocompatibility.

**Figure 1.**
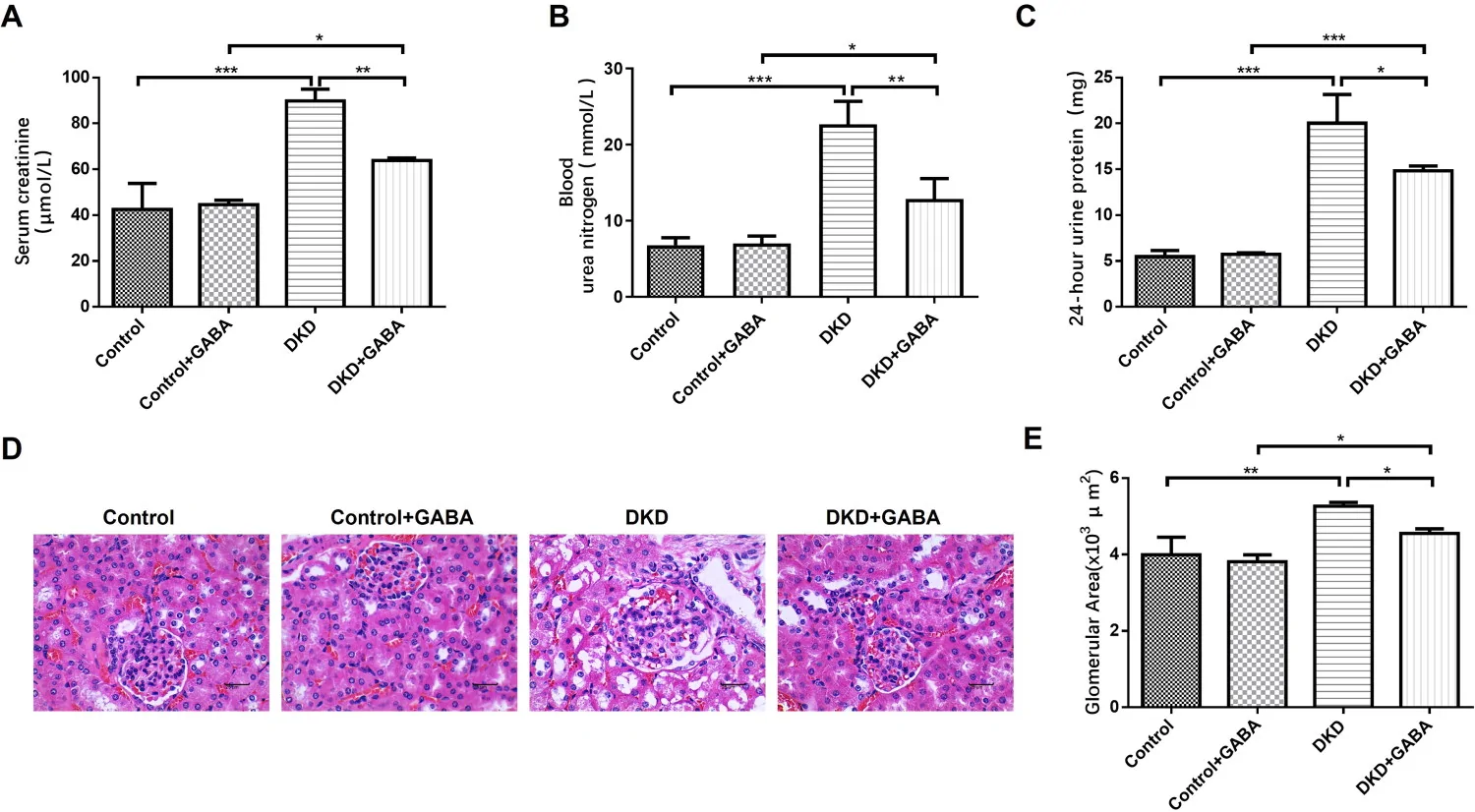
Effects of gamma-aminobutyric acid (GABA) treatment on renal function and structural damage in streptozotocin (STZ)-induced diabetic kidney disease (DKD) mouse model. In control, control + GABA, DKD, and DKD+GABA group (*n* = 5 mice per group), (**A**) serum creatinine (µmol/L), (**B**) blood urea nitrogen (mmol/L) and (**C**) urine protein (mg/24h) levels in serum by enzyme-linked immunosorbent assay (ELISA). (**D**) Representative images of kidney tissue by hematoxylin and eosin (HE) ­staining. (**E**) The statistics of glomerular area (µm^2^). Data are presented as mean ± SD. *P < 0.05, **P < 0.01, ***P < 0.001. Abbreviations: GABA, gamma-aminobutyric acid; STZ, streptozotocin; DKD, diabetic kidney disease; ELISA, enzyme-linked immunosorbent assay; HE, hematoxylin and eosin.

### Effects of GABA treatment on pro-inflammatory factor production in renal tissue and serum of DKD mouse model

Analysis of renal tissues revealed the significant elevation in mRNA levels of pro-inflammatory cytokines (TNF-α, IL-1β, and iNOS) in the DKD group compared to control group, while GABA treatment markedly attenuated these upregulated inflammatory mediators in DKD mice. Similarly, serum levels of these markers were markedly elevated in the DKD group compared to control group, while GABA treatment effectively suppressed the overproduction of these pro-inflammatory cytokines in DKD mice (Figure 2A–F). These findings demonstrated that GABA treatment inhibited pro-inflammatory cytokine expression in STZ-induced DKD mice.

**Figure 2.**
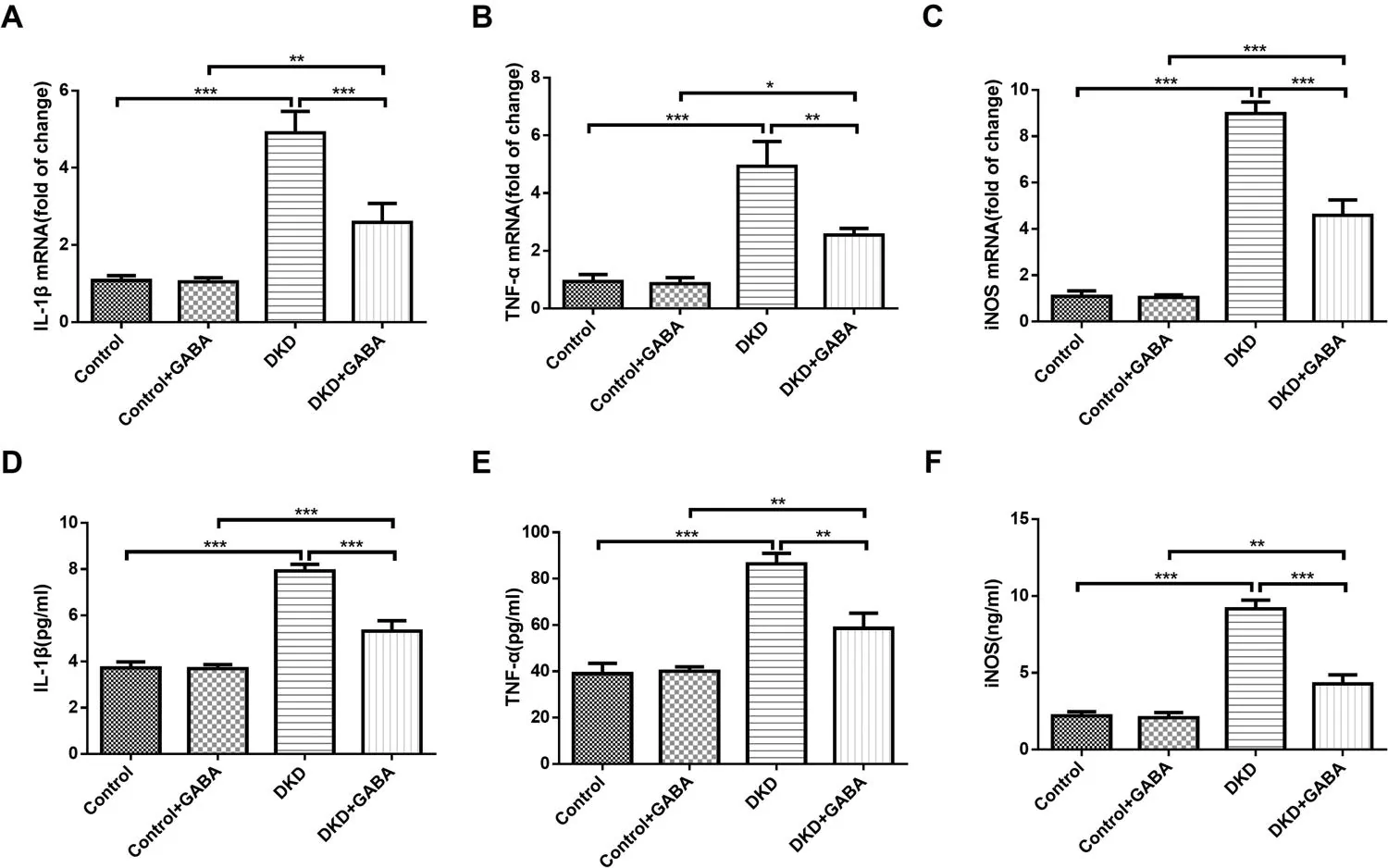
Effects of GABA treatment on pro-inflammatory factor production in renal tissue and serum of DKD mouse model. In control, control + GABA, DKD, and DKD+GABA group (*n* = 5 mice per group), (**A**) interleukin-1β (IL-1β), (**B**) tumor necrosis factor-α (TNF-α), and (**C**) inducible nitric oxide synthase (iNOS) mRNA levels (relative expression) in renal tissues by reverse transcription-quantitative polymerase chain reaction (RT-qPCR). (**D**) IL-1β (pg/mL), (**E**) TNF-α (pg/mL), and (**F**) iNOS (ng/mL) levels in serum by ELISA analysis. Data are presented as mean ± SD. *P < 0.05, **P < 0.01, ***P < 0.001. Abbreviations: GABA, gamma-aminobutyric acid; DKD, diabetic kidney disease; IL-1β, interleukin-1β; TNF-α, tumor necrosis factor-α; iNOS, inducible nitric oxide synthase; RT-qPCR, reverse transcription-quantitative polymerase chain reaction; ELISA, enzyme-linked immunosorbent assay.

### Effects of GABA treatment on macrophage infiltration and polarization

Compared with control group, the expression of CD68 was significantly increased in kidney tissue of the DKD mice, whereas GABA treatment markedly attenuated the increases of CD68^+^ cell in glomerulus (Figure 3A and B). A pronounced shift in macrophage polarization from M2 to M1 was observed in the DKD group, characterized by elevated CD80/CD86 expression and suppressed CD163/CD206 levels (Figure 3C). These findings demonstrated that GABA treatment inhibited the macrophage infiltration and polarization.

**Figure 3.**
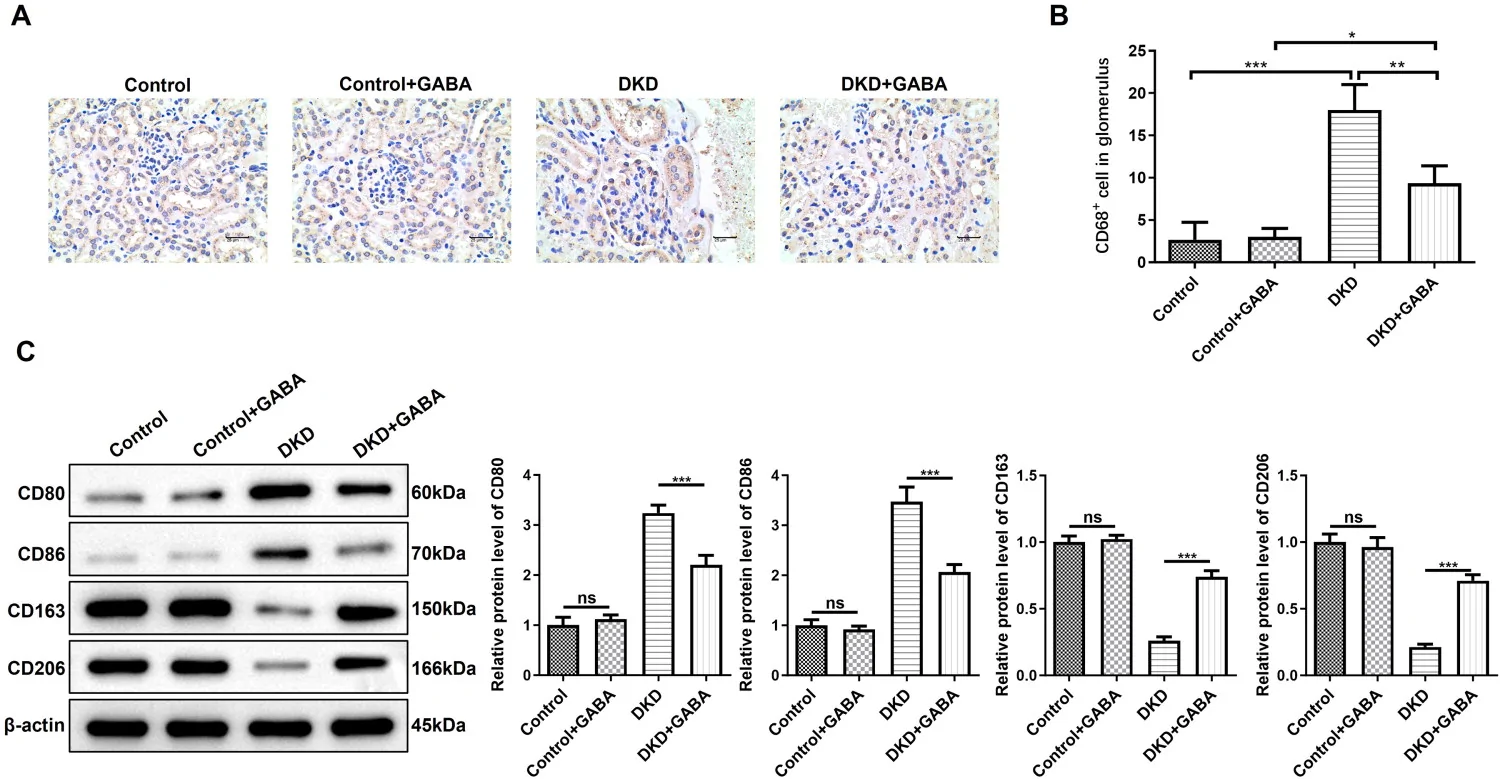
Effects of GABA treatment on macrophage infiltration and polarization. In control, control + GABA, DKD, and DKD+GABA group (*n* = 5 mice per group), (**A**) immunohistochemistry (IHC) analysis of CD68. (**B**) Quantification of CD68^+^ cells per glomerulus. (**C**) CD80, CD86, CD163 and CD206 protein levels in renal tissues by western blot analysis (relative to β-actin). Representative blots are shown; the corresponding β-actin loading controls from the same membranes are provided in the supplementary materials. Data are presented as mean ± SD. *P < 0.05, **P < 0.01, ***P < 0.001. Abbreviations: GABA, gamma-aminobutyric acid; DKD, diabetic kidney disease; IHC, immunohistochemistry; M1, classically activated macrophage; M2, alternatively activated macrophage.

### Effect of GABA treatment on renal tissue apoptosis and podocyte injury in DKD mice

TUNEL assay revealed that apoptotic cells in glomerulis was increased in the DKD group, and the GABA treatment effectively reversed the promoting effect of DKD on renal tissue apoptosis (Figure 4A and B). Concomitant with the phenotypic differentiation of macrophages, the expression levels of both Nephrin and Podocin were significantly downregulated in the DKD group, whereas GABA treatment in DKD mice markedly promoted their expressions (Figure 4C and D). The results indicated that GABA treatment inhibited the renal tissue apoptosis and alleviated podocyte injury in DKD mice.

**Figure 4.**
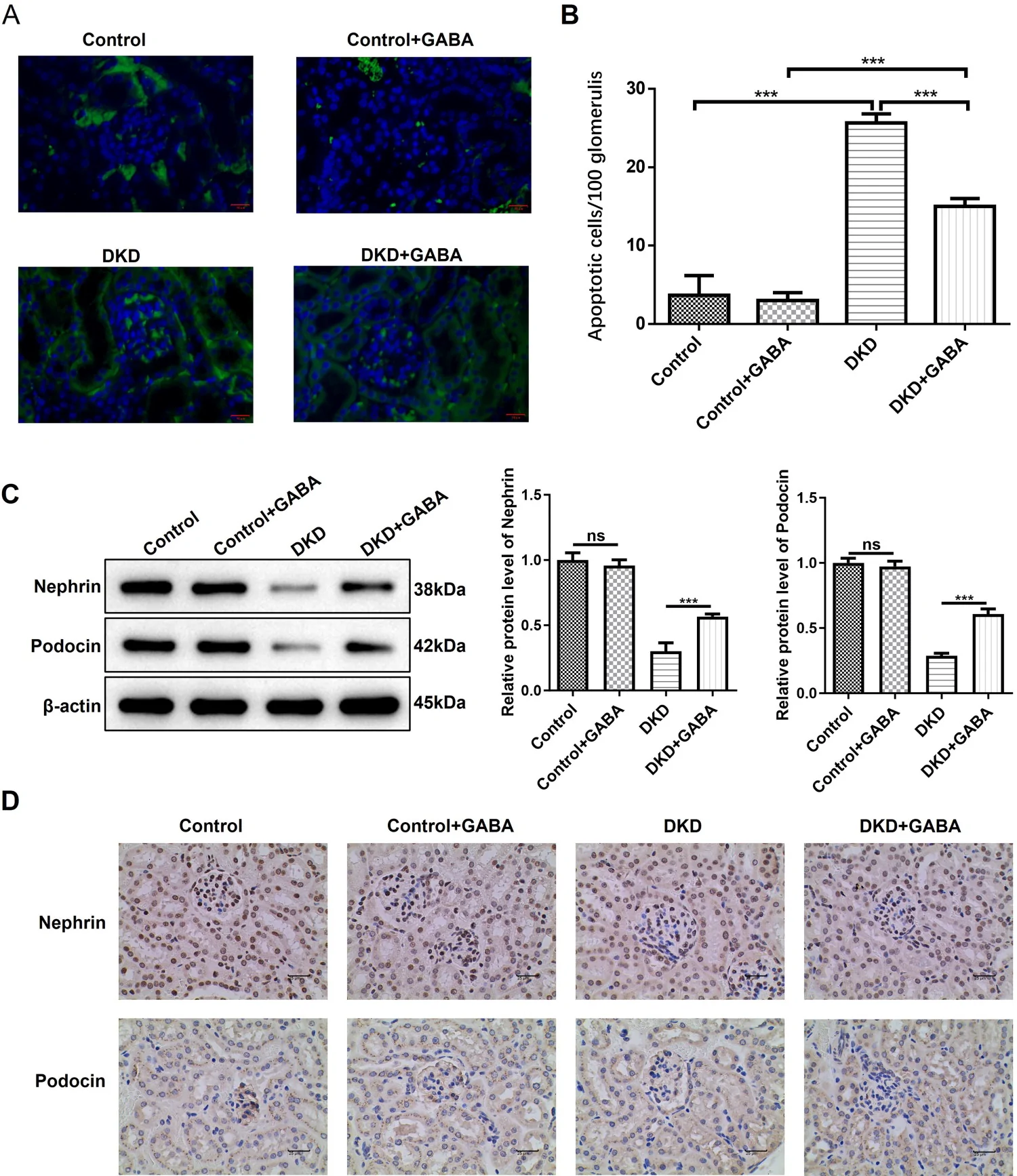
Effect of GABA treatment on renal tissue apoptosis and podocyte injury in DKD mice. In control, control + GABA, DKD, and DKD+GABA group (*n* = 5 mice per group), (**A**) terminal deoxynucleotidyl transferase dUTP nick end-labeling (TUNEL) staining for representative kidney section in mice. (**B**) Apoptotic cells/100 glomerulis. Nephrin and Podocin levels in renal tissues by (**C**) western blot (relative to β-actin) and (**D**) IHC analysis. In (C), representative blots are shown; the corresponding β-actin loading controls from the same membranes are provided in the supplementary materials. Data are presented as mean ± SD. ***P < 0.001. Abbreviations: GABA, gamma-aminobutyric acid; DKD, diabetic kidney disease; TUNEL, terminal deoxynucleotidyl transferase dUTP nick end-labeling; IHC, immunohistochemistry.

### Impact of GABA on high glucose-induced podocyte apoptosis in a macrophage co-culture model

To investigate the effect of macrophage infiltration on podocyte apoptosis, we established a macrophage/podocyte co-culture system in a HG environment. Our experimental results demonstrated that macrophages exposed to HG condition exhibited markedly upregulated iNOS protein expression and downregulated CD206 level compared to those in NG environments, while GABA treatment effectively reversed these alterations; furthermore, the HG-induced mRNA elevations of pro-inflammatory cytokines, including IL-1β, TNF-α, and iNOS were also significantly attenuated by GABA intervention (Figure 5A–D). The apoptosis rates of podocytes in the HG environment significantly increased compared to NG environment, while GABA treatment reduced HG-induced podocyte apoptosis in a macrophage/podocyte co-culture model. Ratio-dependent (1:1 to 4:1 macrophage-to-podocyte ratios) revealed maximal apoptotic susceptibility at the 4:1 ratio under HG conditions (HG+Mφ-4R), whereas GABA intervention significantly reduced the podocyte apoptosis in HG+Mφ-4R + GABA group compared with HG+Mφ-4R group (Figure 5E). This ratio-dependent efficacy suggested GABA modulated macrophage-drived pro-apoptotic process of podocyte in a HG environment.

**Figure 5.**
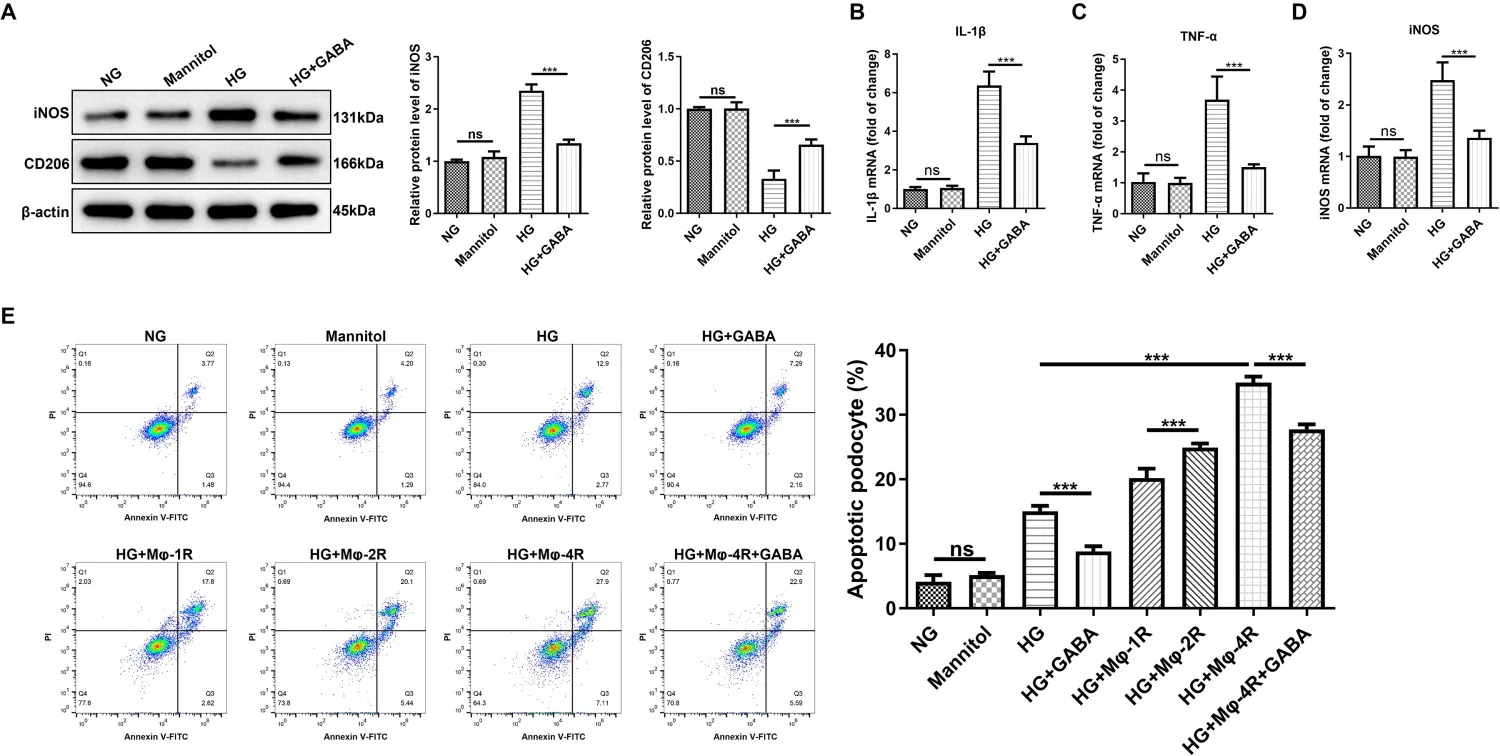
Impact of GABA on high glucose (HG) induced podocyte apoptosis in a macrophage co-culture model. For *in vitro* experiments (n = 3 independent biological replicates): in normal glucose (NG), Mannitol, HG, and HG+GABA group, (**A**) iNOS and CD206 protein levels (relative to β-actin) in macrophage. Representative blots are shown; the corresponding β-actin loading controls from the same membranes are provided in the supplementary materials. (**B**) TNF-α, (**C**) IL-1β, and (**D**) iNOS mRNA levels (relative expression) in macrophage. (**E**) The apoptosis rates (%) in NG, Mannitol, HG, HG+GABA, HG+Mφ-1R (macrophage-to-podocyte ratio 1:1), HG+Mφ-2R (macrophage-to-podocyte ratio 2:1), HG+Mφ-4R (macrophage-to-podocyte ratio 4:1) and HG+Mφ-4R + GABA group. Data are presented as mean ± SD. ***P < 0.001. Abbreviations: GABA, gamma-aminobutyric acid; HG, high glucose; NG, normal glucose; iNOS, inducible nitric oxide synthase; M1, classically activated macrophage; M2, alternatively activated macrophage; TNF-α, tumor necrosis factor-α; IL-1β, interleukin-1β; RT-qPCR, reverse transcription-quantitative polymerase chain reaction; Mφ, macrophage; R: ratio.

### Impact of GABA on JAML expression in renal tissues and podocytes in vivo and in vitro

The STZ-induced diabetic kidney disease environment significantly amplified JAML expression across transcriptional and translational levels in renal tissues, as evidenced by comparative analysis between DKD and control groups by RT-qPCR and IF staining (Figure 6A and 6B). GABA treatment demonstrated therapeutic potential by normalizing JAML protein expression to physiological levels (Figure 6C and 6D). Compared with NG group, JAML mRNA and protein levels were significantly increased in HG environment. GABA treatment inhibited the protein level of JAML in HG environment (Figure 6E-6G). The results indicated that GABA treatment effectively inhibited the high level of JAML in renal tissues of DKD mice and podocytes in HG environment.

**Figure 6.**
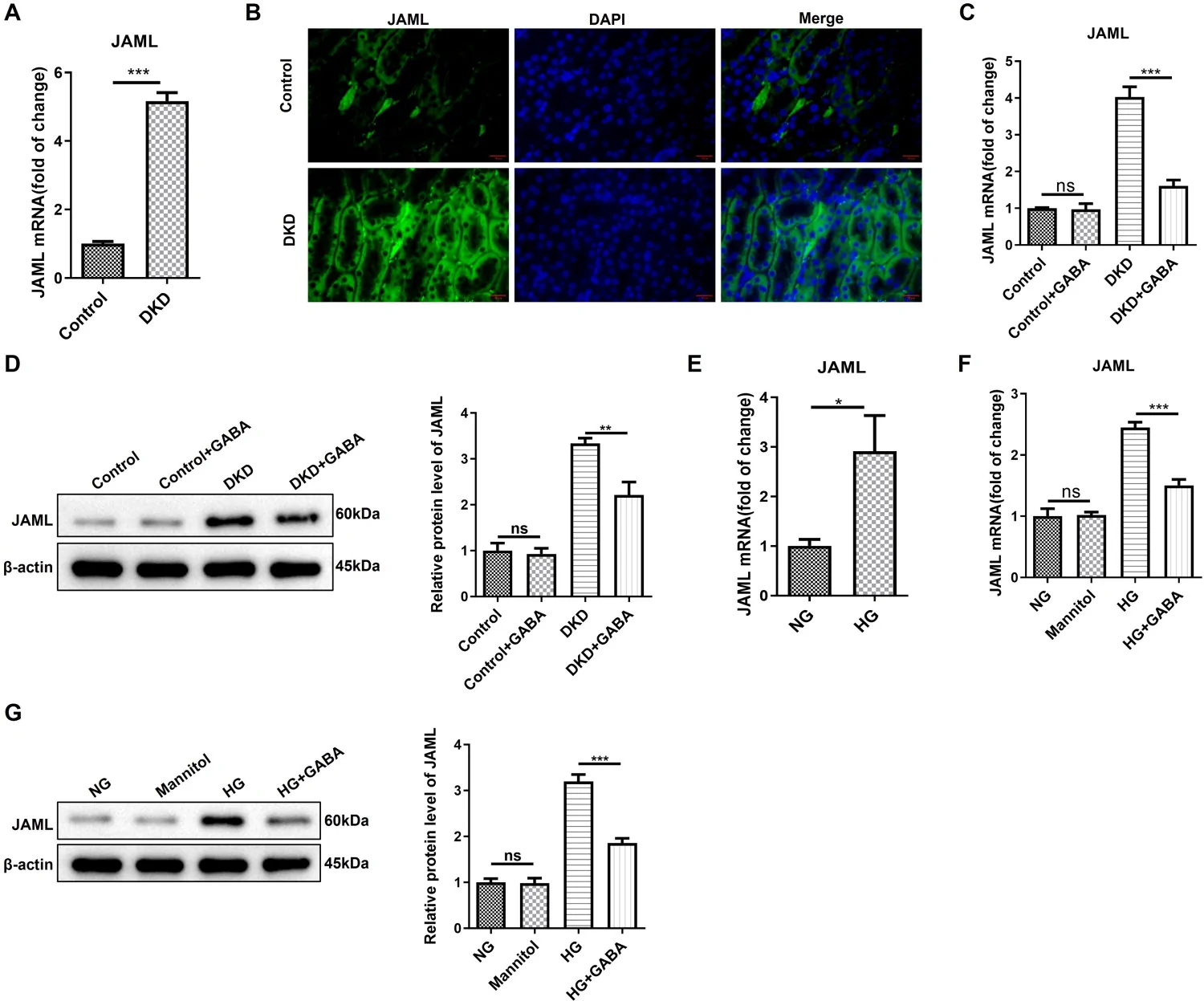
Impact of GABA on junctional adhesion molecule-like protein (JAML) expression in renal tissues and podocytes *in vivo* and *in vitro*. In control and DKD group (*n* = 5 mice per group), JAML mRNA (relative expression) and protein levels by (**A**) RT-qPCR and (**B**) immunofluorescence (IF) analysis. In control, control + GABA, DKD, and DKD+GABA group (*n* = 5 mice per group), JAML mRNA (relative expression) and protein (relative to β-actin) levels by (**C**) RT-qPCR and (**D**) western blot analysis. (**E**) JAML mRNA (relative expression) in NG and HG environment. (**F**) and (**G**) JAML mRNA (relative expression) and protein (relative to β-actin) levels in NG, Mannitol, HG, and HG+GABA group. In (D) and (G), representative blots are shown; the corresponding β-actin loading controls from the same membranes are provided in the supplementary materials. Data are presented as mean ± SD. *P < 0.05, ***P < 0.001. Abbreviations: GABA, gamma-aminobutyric acid; JAML, junctional adhesion molecule-like protein; DKD, diabetic kidney disease; RT-qPCR, reverse transcription-quantitative polymerase chain reaction; IF, immunofluorescence; NG, normal glucose; HG, high glucose.

### The impact of overexpression of JAML on GABA-mediated therapeutic effects in diabetic kidney disease mice

First, JAML overexpression was shown to partially reverse GABA-mediated suppression of renal JAML protein in DKD models, despite GABA’s efficacy in reducing DKD-elevated JAML expression relative to the controls (Figure 7A). Second, we found that the TNF-α, IL-1β, and iNOS in the DKD group were significantly higher than those in control group. GABA treatment effectively suppressed these pro-inflammatory cytokines in DKD group, which was reversed by JAML overexpression (Figure 7B–D). In addition, the DKD-induced up-regulated CD68^+^ cell in glomerulus and glomerular enlargement were significantly inhibited by GABA treatment, while it was reversed by JAML overexpression (Figure 7E–H). It was observed that DKD-induced increased iNOS protein expression, but this effect was inhibited by GABA treatment. However, JAML overexpression dramatically blocked the inhibitory effect of GABA on iNOS expression. On the other hand, the protein levels of CD206 and podocin had opposite consequences compared with iNOS (Figure 7I). TUNEL assay revealed that GABA treatment effectively weakened the promoting effect of DKD on renal tissue apoptosis, which was reversed by JAML overexpression (Figure 7J and K). These results suggested that overexpression of JAML counteracted GABA-mediated therapeutic effects by reversing pro-inflammatory response-driven renal functional deterioration and podocyte injury in DKD mice.

**Figure 7.**
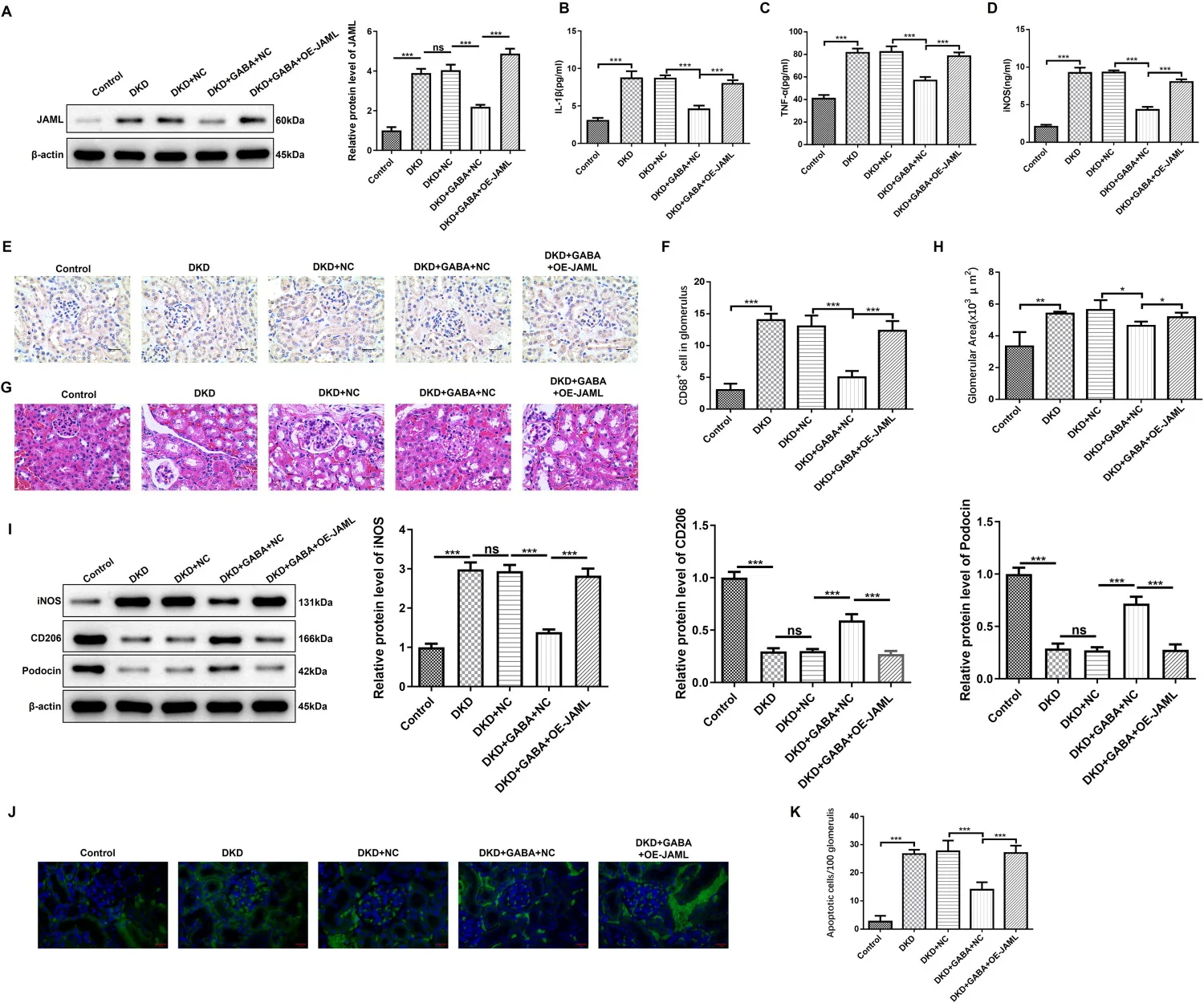
The impact of overexpression of JAML on GABA-mediated therapeutic effects in diabetic kidney disease mice. In control, DKD, DKD+NC, DKD+GABA+NC, and DKD+GABA+OE-JAML group (*n* = 5 mice per group), (**A**) JAML protein levels (relative to β-actin). (**B**) IL-1β (pg/mL), (**C**) TNF-α (pg/mL), and (**D**) iNOS (ng/mL) levels in serum. (**E**) IHC analysis of CD68. (**F**) Quantification of CD68^+^ cells per glomerulus. (**G**) Representative images of kidney tissue by HE staining. (**H**) The statistics of glomerular area (µm^2^). (**I**) iNOS, CD206 and podocin protein levels (relative to β-actin). In (A) and (I), representative blots are shown; the corresponding β-actin loading controls from the same membranes are provided in the supplementary materials. (**J**) TUNEL staining for representative kidney section in mice. (**K**) Apoptotic cells/100 glomerulis. Data are presented as mean ± SD. *P < 0.05, **P < 0.01, ***P < 0.001. Abbreviations: JAML, junctional adhesion molecule-like protein; GABA, gamma-aminobutyric acid; DKD, diabetic kidney disease; NC, negative control; OE-JAML, JAML overexpression; IL-1β, interleukin-1β; TNF-α, tumor necrosis factor-α; iNOS, inducible nitric oxide synthase; ELISA, enzyme-linked immunosorbent assay; IHC, immunohistochemistry; HE, hematoxylin and eosin; TUNEL, terminal deoxynucleotidyl transferase dUTP nick end-labeling.

### JAML regulated excessive macrophage infiltration by mediating FPR2 expression

Through analysis of the ischemia-reperfusion renal injury database (GSE192532), we identified FPR2 as a significantly downregulated gene following JAML knockdown by intersecting differentially expressed genes (DEGs) from normal kidney tissues (JAML knockdown vs. non-knockdown) and IRI model kidney tissues (JAML knockdown vs. non-knockdown). Based on this, we hypothesized that FPR2 played a regulatory role in this mechanism (Figure 8A and B). Co-immunoprecipitation assays revealed a direct interaction between JAML and FPR2 (Figure 8C). FPR2 knockdown abolished the DKD-driven increases in FPR2 and iNOS expression, whereas DKD-induced JAML upregulation unaltered in DKD+KD-FPR2 group. DKD-induced upregulations of JAML, FPR2 and iNOS protein expression were promoted by the overexpression of JAML. The upregulation of FPR2 and iNOS protein expression in DKD+OE-JAML group was abolished by knockdown of FPR2. However, there was no significant change for JAML protein level in DKD+OE-JAML+KD-FPR2 group compared with DKD+OE-JAML+NC group. On the other hand, the protein levels of CD206 and podocin had opposite consequences compared with FPR2 and iNOS (Figure 8D). These results confirmed that FPR2 was the critical role in this regulatory network. DKD-induced up-regulated CD68^+^ cells in glomerulus were significantly inhibited by knockdown of FPR2 or promoted by the overexpression of JAML. And the upregulation of CD68^+^ cells in glomerulus in DKD+OE-JAML group were abolished by knockdown of FPR2 (Figure 8E and F). *In vivo* studies had elucidated that JAML promoted excessive macrophage infiltration by upregulating FPR2 expression.

**Figure 8.**
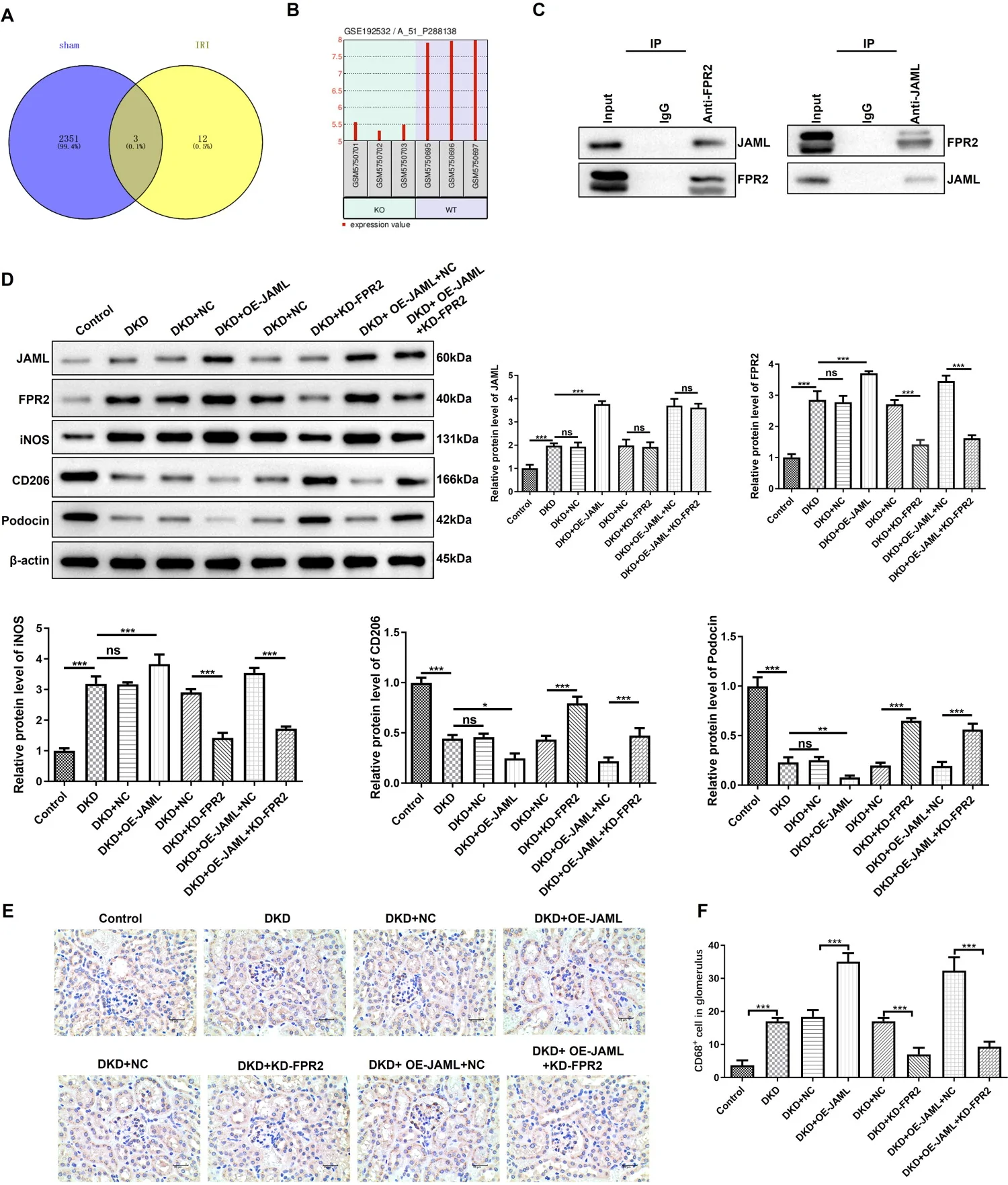
JAML regulated excessive macrophage infiltration by mediating formyl peptide receptor 2 (FPR2) expression. **(A)** Three mRNAs were screened from normal kidney tissues (JAML knockout vs. non- knockout) and ischemia-reperfusion injury (IRI) model kidney tissues (JAML knockout vs. non knockout) **(B)** FPR2 expression in wild-type (WT) and JAML knockout (JAML-KO) by GSE192532 dataset. **(C)** The interaction between JAML and FPR2 by co-immunoprecipitation (Co-IP) assay (*n* = 3 independent experiments). In control, DKD, DKD+negative control (NC), DKD+JAML overexpression (OE-JAML), DKD+FPR2 knockdown (KD-FPR2), DKD+OE-JAML+NC, and DKD+OE-JAML+KD-FPR2 group (*n* = 5 mice per group), **(D)** JAML, FPR2, iNOS, CD206 and podocin protein levels (relative to β-actin). Representative blots are shown; the corresponding β‑actin loading controls from the same membranes are provided in the supplementary materials. (**E**) IHC analysis of CD68. (**F**) Quantification of CD68^+^ cells per glomerulus. Data are presented as mean ± SD. *P < 0.05, **P < 0.01, ***P < 0.001. Abbreviations: JAML, junctional adhesion molecule-like protein; FPR2, formyl peptide receptor 2; DEGs, differentially expressed genes; IRI, ischemia-reperfusion injury; WT, wild-type; JAML-KO, JAML knockout; Co-IP, co-immunoprecipitation; DKD, diabetic kidney disease; NC, negative control; OE-JAML, JAML overexpression; KD-FPR2, FPR2 knockdown; iNOS, inducible nitric oxide synthase; IHC, immunohistochemistry.

## Discussion

The escalating global surge in diabetes prevalence had positioned diabetic kidney disease as the predominant etiology of end-stage renal disease [26]. While conventional therapeutic strategies, including glycemic control, blood pressure management, weight reduction, and lifestyle modification, formed the cornerstone of current clinical practice, their collective efficacy remained suboptimal in halting DKD progression. This therapeutic gap underscored an imperative demand for targeted interventions that addressed the underlying pathophysiological mechanisms driving DKD pathogenesis, thereby offering enhanced clinical outcomes for at-risk populations.

In the present study, we first validated the renoprotective effects of GABA using an STZ-induced DKD mouse model. The *in vivo* results demonstrated that GABA treatment significantly ameliorated diabetes-related renal dysfunction, as evidenced by reduced serum creatinine, blood urea nitrogen, and 24-h urinary protein levels. Histopathological analysis further revealed that GABA alleviated glomerular hypertrophy and mesangial expansion, indicating its reparative effects on renal structural injury. Moreover, GABA markedly suppressed the expression of key pro-inflammatory cytokines (TNF-α, IL-1β, and iNOS) in both renal tissues and serum, and reduced glomerular infiltration of CD68^+^ macrophages along with their polarization toward the pro-inflammatory M1 phenotype. These changes were accompanied by decreased renal apoptosis and restored expression of podocyte markers nephrin and podocin. Collectively, the *in vivo* experiments clearly demonstrate that GABA ameliorates DKD progression by inhibiting inflammation, reducing macrophage infiltration and polarization, attenuating podocyte injury, and diminishing cellular apoptosis, thereby providing robust preclinical evidence for its therapeutic potential.

Podocytes, as highly differentiated cells, formed the structural foundation of the glomerular filtration barrier through their intricate foot processes and slit diaphragms, which were essential for preventing proteinuria and macromolecule leakage while preserving glomerular filtration integrity. Consequently, any disrupt to their structural-functional integrity inevitably led to proteinuria and impaired glomerular filtration [27]. As integral structural scaffolds of the glomerular slit diaphragm, nephrin and podocin formed a dynamic regulatory nexus, where nephrin governed selective permselectivity, while podocin stabilized filtration barrier architecture by maintaining podocyte processed interdigitation and membrane domain specialization [28,29]. The downregulation of podocin and nephrin reflected podocyte foot process effacement and subsequent compromise of glomerular filtration barrier integrity, mechanistically linked to the onset of albuminuria through impaired selective filtration [30,31]. Our findings demonstrated that GABA administration restored nephrin and podocin expression in DKD models, mechanistically linking therapeutic effect of GABA to podocyte preservation. In addition, our experimental data demonstrated that GABA administration markedly attenuated podocyte apoptosis in DKD models, suggesting its potential therapeutic efficacy in preserving glomerular cell viability. In light of previous findings demonstrating that high-glucose directly induced podocyte apoptosis, we employed a macrophage-podocyte co-culture system under high-glucose conditions to investigate the therapeutic potential of GABA in mitigating this pathological process in diabetic kidney disease [32]. Initial observations revealed that HG stimulation induced macrophage polarization imbalance, characterized by upregulated iNOS expression (M1 marker) concomitant with suppressed CD206 expression (M2 marker). Given the dichotomous roles of macrophage subtypes, where pro-inflammatory M1 macrophages secreted IL-6, IL-12, IL-1β, TNF-α and expressed iNOS, whereas anti-inflammatory M2 suppressed these inflammatory mediators [12], our data demonstrated HG preferentially drived M1-dominant activation. Crucially, quantitative analysis established a direct correlation between macrophage infiltration and podocyte apoptosis under HG conditions, and podocyte apoptosis and M1-dominant activation were significantly mitigated by GABA intervention. These findings collectively suggest GABA exerted podocyte-protective effects through modulation of macrophage polarization, potentially *via* suppression of M1-mediated inflammatory cascades.

The emerging role of JAML in renal pathophysiology warrants focused investigation, particularly given its dual functionality as both an inflammatory mediator and a structural regulator in kidney injury [33]. It was reported that podocyte injury and proteinuria were significantly ameliorated in two distinct diabetic murine models through podocyte-specific JAML l deletion [34]. Notably, in our study, the observed upregulation of JAML in DKD models correlated with disrupted podocyte slit diaphragm architecture, suggesting its involvement in pathways critical for maintaining filtration barrier integrity. In addition, FPR2 knockout reversed DKD-induced FPR2 upregulation but unabled to change JAML overexpression in DKD-mice, establishing that JAML was an upstream regulator of FPR2. The effect of downstream regulatory factors of JAML on its function in cancers or diseases had been extensively investigated. For instance, JAML mediated the PI3K-AKT-mTOR pathway in colorectal cancer, Wnt/β-catenin signaling in lung adenocarcinoma, p38 signaling in gastric cancer to regulate tumor progression [35–37].

FPR2, a chemoattractant receptor of the FPR family, and its murine homolog Fpr2 were highly expressed in macrophages/monocytes and neutrophils, where they engaged diverse peptide and lipid ligands to exert context-dependent pro- or anti-inflammatory effects through ligand-specific activation of distinct signaling pathways [38,39]. The study revealed the regulation patterns of FPR2 during macrophage polarization, with its expression being markedly upregulated in pro-inflammatory M1 differentiation while suppressed in anti-inflammatory M2 differentiation, establishing FPR2 as a novel biomarker for identifying M1 macrophages [40]. FPR2 knockout conferred protection against nutritional insulin resistance by coordinately controlling metabolic parameters by reducing weight gain and immunological components, inhibiting macrophage chemotaxis and M1 polarization-mediated inflammation [41]. Inhibition of FPR2 ameliorated M1 macrophage polarization [42]. Our study found that the knockdown of FPR2 in DKD-mice suppressed iNOS expression (M1 marker) concomitant upregulated with CD206 expression (M2 marker) compared with the DKD group, suggesting that the knockdown of FPR2 ameliorated M1 macrophage polarization.

In addition to the pivotal role of TNF-α itself, recent clinical and translational studies have highlighted the prognostic significance of circulating soluble TNF receptors (sTNFR1 and sTNFR2) in DKD [43]. Elevated levels of these receptors are strongly associated with the progression of renal function decline, increased risk of end-stage renal disease, and all-cause mortality in diabetic patients, often exhibiting superior predictive value over TNF-α alone [44,45]. While our current study primarily focused on the upstream JAML/FPR2 axis in modulating macrophage-driven inflammation and podocyte injury, this axis may represent a novel regulatory layer influencing the TNF-α/TNFR signaling pathway or the bioavailability of its soluble receptors. Future investigations exploring the interplay between the JAML/FPR2 mechanism and the TNF receptor system could provide deeper insights into the inflammatory network driving DKD progression.

In summary, our findings demonstrated that GABA ameliorated diabetic kidney disease-induced podocyte injury *via* JAML-FPR2 axis to suppress the macrophage infiltration. These results indicated that the inhibition of FPR2 might be a promising therapeutic strategy against diabetic kidney disease.

## Supplementary Material

Western Blot.docx

## Data Availability

The data that support the findings of this study are available from the corresponding authors, AHZ and HPN, upon reasonable request.

## References

[1] Cheng W, Wang C, Ma M, et al. Hydroxyurea mitigates diabetic kidney disease through mTOR-S6K signaling pathway in STZ-induced diabetic mice. Front Cell Dev Biol. 2025;13:1529901. doi: 10.3389/fcell.2025.1529901. PMC11794838 39911324

[2] Xiang E, Han B, Zhang Q, et al. Human umbilical cord-derived mesenchymal stem cells prevent the progression of early diabetic nephropathy through inhibiting inflammation and fibrosis. Stem Cell Res Ther. 2020;11(1):336. doi: 10.1186/s13287-020-01852-y. PMC7397631 32746936

[3] Hung PH, Hsu YC, Chen TH, et al. Recent advances in diabetic kidney diseases: from kidney injury to kidney fibrosis. Int J Mol Sci. 2021;22(21):11857. doi: 10.3390/ijms222111857. PMC8584225 34769288

[4] Jiang WJ, Xu CT, Du CL, et al. Tubular epithelial cell-to-macrophage communication forms a negative feedback loop via extracellular vesicle transfer to promote renal inflammation and apoptosis in diabetic nephropathy. Theranostics. 2022;12(1):324–339. doi: 10.7150/thno.63735. PMC8690920 34987648

[5] Mora C, Navarro JF. Inflammation and pathogenesis of diabetic nephropathy. Metabolism. 2004;53(2):265–266. author reply 266-267. doi: 10.1016/j.metabol.2003.11.005. 14767884

[6] Xu C, Chen Y, Liu Z, et al. Hedysarum polybotrys polysaccharide attenuates renal inflammatory infiltration and fibrosis in diabetic mice by inhibiting the HMGB1/RAGE/TLR4 pathway. Exp Ther Med. 2023;26(4):493. doi: 10.3892/etm.2023.12192. PMC10523352 37771649

[7] Man X, Wang L, Liao H, et al. Capilliposide A attenuates diabetic nephropathy via modulation of NF-κB/TLR4 and apoptotic pathways. Sci Rep. 2025;15(1):31478. doi: 10.1038/s41598-025-07066-7. PMC12381292 40858660

[8] Hasan IH, Alqahtani QH, Sarawi WS, et al. Upregulation of Nrf2/HO-1 signaling and farnesoid X receptor and attenuation of oxidative stress and inflammation mediate the protective effect of sitagliptin against diabetic nephropathy in rats. Int Immunopharmacol. 2025;163:115260. doi: 10.1016/j.intimp.2025.115260. 40700832

[9] Ma J, Chadban SJ, Zhao CY, et al. TLR4 activation promotes podocyte injury and interstitial fibrosis in diabetic nephropathy. PLoS One. 2014;9(5):e97985. doi: 10.1371/journal.pone.0097985. PMC4026484 24842252

[10] Lv J, Chen Q, Shao Y, et al. Cross-talk between angiotensin-II and toll-like receptor 4 triggers a synergetic inflammatory response in rat mesangial cells under high glucose conditions. Biochem Biophys Res Commun. 2015;459(2):264–269. doi: 10.1016/j.bbrc.2015.02.096. 25732086

[11] Wynn TA, Barron L. Macrophages: master regulators of inflammation and fibrosis. Semin Liver Dis. 2010;30(3):245–257. doi: 10.1055/s-0030-1255354. PMC2924662 20665377

[12] Tang PM, Nikolic-Paterson DJ, Lan HY. Macrophages: versatile players in renal inflammation and fibrosis. Nat Rev Nephrol. 2019;15(3):144–158. doi: 10.1038/s41581-019-0110-2. 30692665

[13] Abudoureyimu A, Chen C, Hu Y, et al. Quercetin alleviates diabetic nephropathy by inhibiting M1 macrophage polarization via targeting NLRC5/NLRP3 pathway. Cell Immunol. 2025;414:104997. doi: 10.1016/j.cellimm.2025.104997. 40570646

[14] Wang Y, Wang H, Li Y. Protocatechuic aldehyde ameliorates high glucose-induced podocyte injury by attenuating inflammation, oxidative stress, and apoptosis via suppression of endoplasmic reticulum stress through the GSK3β/Nrf2 pathway. Front Cell Dev Biol. 2025;13:1693955. doi: 10.3389/fcell.2025.1693955. PMC12645221 41306290

[15] Tian J, Dang H, Kaufman DL. Combining antigen-based therapy with GABA treatment synergistically prolongs survival of transplanted ß-cells in diabetic NOD mice. PLoS One. 2011;6(9):e25337. doi: 10.1371/journal.pone.0025337. PMC3178649 21966502

[16] Tian J, Yong J, Dang H, et al. Oral GABA treatment downregulates inflammatory responses in a mouse model of rheumatoid arthritis. Autoimmunity. 2011;44(6):465–470. doi: 10.3109/08916934.2011.571223. PMC5787624 21604972

[17] Son M, Oh S, Lee HS, et al. Gamma-aminobutyric acid-salt attenuated high cholesterol/high salt diet induced hypertension in mice. Korean J Physiol Pharmacol. 2021;25(1):27–38. doi: 10.4196/kjpp.2021.25.1.27. PMC7756537 33361535

[18] Daems C, Welsch S, Boughaleb H, et al. Early Treatment with Empagliflozin and GABA Improves β-Cell Mass and Glucose Tolerance in Streptozotocin-Treated Mice. J Diabetes Res. 2019;2019:2813489. doi: 10.1155/2019/2813489. PMC6701376 31467926

[19] Su R, Chang L, Zhou T, et al. Effects of GABA on Oxidative Stress and Metabolism in High-Glucose Cultured Mongolian Sheep Kidney Cells. Int J Mol Sci. 2024;25(18):10033. doi: 10.3390/ijms251810033. PMC11432592 39337519

[20] Ebnet K. Junctional Adhesion Molecules (JAMs): cell adhesion receptors with pleiotropic functions in cell physiology and development. Physiol Rev. 2017;97(4):1529–1554. doi: 10.1152/physrev.00004.2017. 28931565

[21] Lauko A, Mu Z, Gutmann DH, et al. Junctional adhesion molecules in cancer: a paradigm for the diverse functions of cell-cell interactions in tumor progression. Cancer Res. 2020;80(22):4878–4885. doi: 10.1158/0008-5472.CAN-20-1829. PMC7669553 32816855

[22] Witherden DA, Verdino P, Rieder SE, et al. The junctional adhesion molecule JAML is a costimulatory receptor for epithelial gammadelta T cell activation. Science. 2010;329(5996):1205–1210. doi: 10.1126/science.1192698. PMC2943937 20813954

[23] Huang W, Wang BO, Hou YF, et al. JAML promotes acute kidney injury mainly through a macrophage-dependent mechanism. JCI Insight. 2022;7(14):e158571. doi: 10.1172/jci.insight.158571. PMC9431718 35708906

[24] Cao Y, Hao Y, Li H, et al. Role of endoplasmic reticulum stress in apoptosis of differentiated mouse podocytes induced by high glucose. Int J Mol Med. 2014;33(4):809–816. doi: 10.3892/ijmm.2014.1642. PMC3976130 24503896

[25] Han J, Pang X, Zhang Y, et al. Hirudin protects against kidney damage in streptozotocin-induced diabetic nephropathy rats by inhibiting inflammation via P38 MAPK/NF-κB pathway. Drug Des Devel Ther. 2020;14:3223–3234. doi: 10.2147/DDDT.S257613. PMC7425656 32848363

[26] Huang F, Wang Q, Guo F, et al. FoxO1-mediated inhibition of STAT1 alleviates tubulointerstitial fibrosis and tubule apoptosis in diabetic kidney disease. EBioMedicine. 2019;48:491–504. doi: 10.1016/j.ebiom.2019.09.002. PMC6838438 31629675

[27] Tian X, Ishibe S. Targeting the podocyte cytoskeleton: from pathogenesis to therapy in proteinuric kidney disease. Nephrol Dial Transplant. 2016;31(10):1577–1583. doi: 10.1093/ndt/gfw021. PMC5039341 26968197

[28] Audzeyenka I, Rachubik P, Rogacka D, et al. Cathepsin C is a novel mediator of podocyte and renal injury induced by hyperglycemia. Biochim Biophys Acta Mol Cell Res. 2020;1867(8):118723. doi: 10.1016/j.bbamcr.2020.118723. 32302668

[29] Zhang C, Zhu X, Li L, et al. A small molecule inhibitor MCC950 ameliorates kidney injury in diabetic nephropathy by inhibiting NLRP3 inflammasome activation. Diabetes Metab Syndr Obes. 2019;12:1297–1309. doi: 10.2147/DMSO.S199802. PMC6684489 31447572

[30] Huber TB, Benzing T. The slit diaphragm: a signaling platform to regulate podocyte function. Curr Opin Nephrol Hypertens. 2005;14(3):211–216. doi: 10.1097/01.mnh.0000165885.85803.a8. 15821412

[31] Qi XM, Wang J, Xu XX, et al. FK506 reduces albuminuria through improving podocyte nephrin and podocin expression in diabetic rats. Inflamm Res. 2016;65(2):103–114. doi: 10.1007/s00011-015-0893-y. PMC4712236 26566632

[32] Qian X, Tan J, Liu L, et al. MicroRNA-134-5p promotes high glucose-induced podocyte apoptosis by targeting bcl-2. Am J Transl Res. 2018;10(3):989–997. PMC5883139 29636888

[33] Fornoni A, Merscher S. Lipid metabolism gets in a JAML during kidney disease. Cell Metab. 2020;32(6):903–905. doi: 10.1016/j.cmet.2020.11.002. PMC7978490 33264600

[34] Fu Y, Sun Y, Wang M, et al. Elevation of JAML promotes diabetic kidney disease by modulating podocyte lipid metabolism. Cell Metab. 2020;32(6):1052–1062.e8. e1058. doi: 10.1016/j.cmet.2020.10.019. 33186558

[35] Fang Y, Liu Y, Dong Z, et al. JAML overexpressed in colorectal cancer promotes tumour proliferation by activating the PI3K-AKT-mTOR signalling pathway. Sci Rep. 2024;14(1):24514. doi: 10.1038/s41598-024-75180-z. PMC11489459 39424882

[36] Fang Y, Yang J, Zu G, et al. Junctional adhesion molecule-like protein promotes tumor progression and metastasis via p38 signaling pathway in gastric cancer. Front Oncol. 2021;11:565676. doi: 10.3389/fonc.2021.565676. PMC7991718 33777731

[37] Wu Q, Li R, Wang QX, et al. Junctional adhesion molecule-like protein promotes tumor progression via the Wnt/β-catenin signaling pathway in lung adenocarcinoma. J Transl Med. 2022;20(1):260. doi: 10.1186/s12967-022-03457-w. PMC9171988 35672776

[38] Cooray SN, Gobbetti T, Montero-Melendez T, et al. Ligand-specific conformational change of the G-protein-coupled receptor ALX/FPR2 determines proresolving functional responses. Proc Natl Acad Sci U S A. 2013;110(45):18232–18237. doi: 10.1073/pnas.1308253110. PMC3831442 24108355

[39] Ye RD, Boulay F, Wang JM, et al. International Union of Basic and Clinical Pharmacology. LXXIII. Nomenclature for the formyl peptide receptor (FPR) family. Pharmacol Rev. 2009;61(2):119–161. doi: 10.1124/pr.109.001578. PMC2745437 19498085

[40] Jablonski KA, Amici SA, Webb LM, et al. Novel markers to delineate Murine M1 and M2 Macrophages. PLoS One. 2015;10(12):e0145342. doi: 10.1371/journal.pone.0145342. PMC4689374 26699615

[41] Chen X, Zhuo S, Zhu T, et al. Fpr2 deficiency alleviates diet-induced insulin resistance through reducing body weight gain and inhibiting inflammation mediated by macrophage chemotaxis and M1 polarization. Diabetes. 2019;68(6):1130–1142. doi: 10.2337/db18-0469. PMC6905484 30862681

[42] Xie K, Zhang Y, Ou X, et al. Taurine ameliorates liver fibrosis by repressing Fpr2-regulated macrophage M1 polarization. Eur J Pharmacol. 2025;997:177614. doi: 10.1016/j.ejphar.2025.177614. 40216178

[43] Moroney E, Niewczas MA, Ekinci EI, et al. Soluble Tumor Necrosis Factor Receptors (sTNFRs) as biomarkers for diabetic kidney disease. J Diabetes Complications. 2026;40(1):109204. doi: 10.1016/j.jdiacomp.2025.109204. PMC13091037 41175629

[44] Murakoshi M, Gohda T, Suzuki Y. Circulating tumor necrosis factor receptors: a potential biomarker for the progression of diabetic kidney disease. Int J Mol Sci. 2020;21(6):1957. doi: 10.3390/ijms21061957. PMC7139523 32183005

[45] Scurt FG, Menne J, Brandt S, et al. Endostatin, soluble tumour necrosis factor receptor 1 and soluble tumour necrosis factor receptor 2 cannot predict new onset of microalbuminuria in patients with type 2 diabetes. Diabetes Metab Res Rev. 2024;40(3):e3753. doi: 10.1002/dmrr.3753. 38050450

