# Supplementary material for: GABA ameliorates diabetic kidney disease-induced podocyte injury via JAML-FPR2 axis-mediated suppression of macrophage infiltration
Source: Ren Fail. 2026 Oct 1;48(1):2736906. doi: 10.1080/0886022X.2026.2736906 (PMC13637784; doi:10.1080/0886022X.2026.2736906)
Supplement: Supplementary file 1 [file IRNF_A_2736906_SM6821.docx]

Fig. 3C CD80 and its corresponding β-actin

Fig. 3C CD86 and its corresponding β-actin

Fig. 3C CD163 and its corresponding β-actin

Fig. 3C CD206 and its corresponding β-actin

Fig. 3C β-actin

Fig. 4C Nephrin and its corresponding β-actin

Fig. 4C Podocin and its corresponding β-actin

Fig. 4C β-actin

Fig. 5A iNOS and its corresponding β-actin

Fig. 5A CD206 and its corresponding β-actin

Fig. 5A β-actin

Fig. 6D JAML and its corresponding β-actin

Fig. 6D β-actin

Fig. 6G JAML and its corresponding β-actin

Fig. 6G β-actin

Fig. 7A JAML and its corresponding β-actin

Fig. 7A β-actin

Fig. 7I iNOS and its corresponding β-actin

Fig. 7I CD206 and its corresponding β-actin

Fig. 7I Podocin and its corresponding β-actin

Fig. 7I β-actin

Fig. 8C Anti-FPR2 FPR2

Fig. 8C Anti-FPR2 JAML

Fig. 8C Anti-JAML FPR2

Fig. 8C Anti-JAML JAML

Fig. 8D JAML and its corresponding β-actin

Fig. 8D FPR2 and its corresponding β-actin

Fig. 8D iNOS and its corresponding β-actin

Fig. 8D CD206 and its corresponding β-actin

Fig. 8D Podocin and its corresponding β-actin

Fig. 8D β-actin
